# Human genetics of metabolic dysfunction–associated steatotic liver disease: from variants to cause to precision treatment

**DOI:** 10.1172/JCI186424

**Published:** 2025-04-01

**Authors:** Vincent L. Chen, Annapurna Kuppa, Antonino Oliveri, Yanhua Chen, Prabhu Ponnandy, Puja B. Patel, Nicholette D. Palmer, Elizabeth K. Speliotes

**Affiliations:** 1 Division of Gastroenterology and Hepatology, Department of Internal Medicine, University of Michigan, Ann Arbor, Michigan, USA.; 2 Department of Biochemistry, Wake Forest University School of Medicine, Winston-Salem, North Carolina, USA.; 3 Department of Computational Medicine and Bioinformatics, University of Michigan, Ann Arbor, Michigan, USA.

## Abstract

Metabolic dysfunction–associated steatotic liver disease (MASLD) is characterized by increased hepatic steatosis with cardiometabolic disease and is a leading cause of advanced liver disease. We review here the genetic basis of MASLD. The genetic variants most consistently associated with hepatic steatosis implicate genes involved in lipoprotein input or output, glucose metabolism, adiposity/fat distribution, insulin resistance, or mitochondrial/ER biology. The distinct mechanisms by which these variants promote hepatic steatosis result in distinct effects on cardiometabolic disease that may be best suited to precision medicine. Recent work on gene-environment interactions has shown that genetic risk is not fixed and may be exacerbated or attenuated by modifiable (diet, exercise, alcohol intake) and nonmodifiable environmental risk factors. Some steatosis-associated variants, notably those in patatin-like phospholipase domain-containing 3 (*PNPLA3*) and transmembrane 6 superfamily member 2 (*TM6SF2*), are associated with risk of developing adverse liver-related outcomes and provide information beyond clinical risk stratification tools, especially in individuals at intermediate to high risk for disease. Future work to better characterize disease heterogeneity by combining genetics with clinical risk factors to holistically predict risk and develop therapies based on genetic risk is required.

## Introduction

Metabolic dysfunction–associated steatotic liver disease (MASLD) is characterized by excess hepatic triglyceride content with cardiometabolic disease and encompasses a spectrum of disease that includes deposition of fat in the liver (steatosis), inflammation associated with that fat (steatohepatitis; metabolic dysfunction–associated steatohepatitis [MASH]), liver (fibrosis), and extensive fibrosis with nodular regeneration (cirrhosis) (1, 2). Cirrhosis can progress to hepatic decompensation and hepatocellular carcinoma (HCC) and was the ninth leading cause of death in the US in 2022 (3). MASLD is 25%–50% heritable, and the genetic factors that predispose to this disease have been explored (4–6).

Here, we review phenotypes that have been used to carry out genome-wide association studies (GWAS) and identify loci associated with MASLD. We discuss how combining genetic variants into polygenic risk scores (PRS) can identify people at risk of MASLD and advanced liver disease. Finally, we speculate on what the future of MASLD treatment could look like in the era of precision medicine.

## Phenotypes

Table 1 summarizes the advantages and disadvantages of various measures of hepatic steatosis. Traditionally, the reference standard of MASLD diagnosis was liver biopsy. However, it is an invasive procedure that carries risk of complications including pain, bleeding, and biliary injury. Liver biopsy is also limited by sampling error (7), limited inter-rater reliability (8), and high cost (9).

Noninvasive measures of hepatic steatosis are increasingly being used both in clinical practice and for research. The accurate noninvasive metric of steatosis is imaging. MRI proton density fat fraction (MRI-PDFF) is the most accurate imaging method to quantify hepatic steatosis, with sensitivity and specificity reported at greater than 90% for distinguishing any steatosis (≥5%) from no steatosis (<5%) (10). CT measurements of steatosis (usually defined as lower liver attenuation relative to spleen or a “phantom” control) is another measure that is highly sensitive and specific (>80% for both) for severe steatosis (>30%) (11, 12), but less accurate for measuring mild hepatic steatosis. CT is more widely available than MRI-PDFF, but involves ionizing radiation. Perhaps the most used and least expensive imaging modality approach in clinical practice is ultrasound, which uses hepatic echogenicity, vascular blurring, or subcutaneous tissue thickening for detection. Ultrasound is similar in sensitivity and specificity to CT for detection of severe steatosis but is highly operator dependent with low inter- and intra-rater reliability and can be technically challenging in patients with large body habitus (13). Compared with biopsy, all imaging modalities measure steatosis in a large portion of the liver (9), but may be influenced by other deposits in the liver (14). For example, increased hepatic echogenicity on ultrasound can be caused by fat but also by iron. Decreased attenuation on CT can also be caused by decreased levels of glycogen, iron, or copper content.

Blood-based laboratory tests are increasing in popularity as a measure of steatosis because they are even less expensive and more widely available than imaging studies. Some investigators have used chronic alanine aminotransferase (ALT) elevations in the absence of a competing etiology of liver disease to define MASLD (15). This definition has demonstrated high specificity (>85%) (16, 17), though undiagnosed non-MASLD liver diseases also increase ALT (18). Others have utilized complex laboratory indices of hepatic steatosis, such as fatty liver index (19), hepatic steatosis index (20), Framingham steatosis index (21), and Dallas steatosis index (22), which have all demonstrated moderate to high sensitivity and specificity for hepatic steatosis. However, these are indirect measures which include not only liver enzyme levels, but also age, sex, BMI, and/or diabetes status. Therefore, when applied in the general population, genetic studies on these scores have a high probability of identifying traits correlated with steatosis, such as obesity, waist-hip ratio, and diabetes. Herein, we do not consider variants that only associate with laboratory tests as MASLD variants unless they also associate with an imaging or histologic measure of steatosis.

Finally, International Classification of Diseases (ICD) codes have been used to define hepatic steatosis (23). These codes are useful when analyzing large insurance claims or national healthcare databases where more granular data including imaging results or even laboratory values are not available. These codes have relatively high specificity for MASLD (>80-90%), but sensitivity is less than 50% in most studies, presumably because many patients who have hepatic steatosis remain undiagnosed (17).

## GWAS of MASLD

Several studies (5, 15, 18, 24–42) (summarized in Table 2) with genome-wide coverage, e.g., array or sequencing, have assessed genetic associations with hepatic steatosis using imaging and/or histology as a discovery and/or validation phenotype.

Unbiased evaluations of the genome have provided novel insight into the genomic architecture of MASLD. The earliest work in this area came out of the Dallas Heart Study using a custom nonsynonymous high density array to assess association with hepatic triglyceride (TG) content using proton magnetic resonance spectroscopy (40). A missense mutation, I148M(rs738409), was identified in the patatin-like phospholipase domain-containing 3 gene (*PNPLA3*) and was associated with increased hepatic fat. Differences in allele frequency in Hispanic-, European- and African-American individuals aligned with prevalence differences in these groups. PNPLA3 is a critical regulator of lipid metabolism in the liver and is expressed on lipid droplets (43, 44). Beyond its reproducible association with MASLD (15, 18, 25, 27–30, 32–35, 37–41), *PNPLA3* I148M has been recognized as a risk factor for steatohepatitis, fibrosis/cirrhosis, and HCC (5, 25, 45–48).

Subsequently, more comprehensive efforts were undertaken to capture the contribution of common genetic variation, both coding and noncoding, to disease predisposition. In 2011, Speliotes et al. (5) performed a metaanalysis of four population-based European-ancestry studies to assess the association of approximately 2.4 million variants with MASLD as assessed by CT. In addition to replicating effects at *PNPLA3*, their results showed suggestive lysophospholipase-like 1 gene, glucokinase regulator gene (*GCKR*), and transmembrane 6 superfamily member 2 gene (*TM6SF2*; labeled as the nearest gene neurocan [*NCAN*]) associations. *TM6SF2* was also identified a few years later on exome-wide analysis (34). These results were then extended to provide generalizability across diverse race/ethnic cohorts (4, 29, 33). The protein phosphatase 1 regulatory subunit 3B gene (*PPP1R3B*), which associated with liver attenuation, did not associate with MASLD histology (5) and was subsequently shown to promote liver glycogen storage, as opposed to hepatic steatosis, both of which affect liver attenuation (49).

With the advent of biobanks, investigators have been able to bolster sample size and resultant power to detect associations with MASLD. An exome-wide association study of ALT in 46,544 individuals based on whole-exome sequencing with validation for ICD code-diagnosed MASLD identified a splice variant in the hydroxysteroid 17-β dehydrogenase 13 gene (*HSD17B13*) that was associated with protection from liver disease (24). This variant was validated in histologic MASLD and associated with reduced likelihood of steatohepatitis (24). Around the same time, another group independently reported a different variant in *HSD17B13,* in high-linkage disequilibrium with the splice variant, based on a candidate gene analysis and found it was associated with greater hepatic steatosis, but consistent with the other study, decreased steatohepatitis, as well as a trend toward reduced fibrosis (50). Another multiancestry GWAS study also reported *HSD17B13* to associate with MASLD at genome-wide significance levels (15).

Anstee et al. (25) used a histology cohort of 1,483 European MASLD cases and 17,781 matched controls to identify contributors to MASLD. Their findings supported the association of *PNPLA3*, *TM6SF2,* and *GCKR*, although *GCKR* did not replicate, and additionally identified *HSD17B13*. Consistent with previous reports (24, 50), variation at *HSD17B13* was associated with protection from MASLD and links to research describing decreased levels of 13-cis and all-trans retinoic acid in human livers with MASLD (51).

Parisinos et al. (39) performed a GWAS in UK Biobank to identify variants associated with liver MRI-PDFF. Among 14,440 European individuals, four loci were genome-wide significant, including the apolipoprotein E gene (*APOE*). This finding was also supported by findings from the Genetics of Obesity-related Liver Disease (GOLD) Consortium, with a meta-analysis of eight multiethnic population-based cohorts with CT-measured liver attenuation (38). Phenome-wide association analyses (PheWAS) suggested significant pleiotropy at this locus, i.e., increased hepatic steatosis also associated with lower cholesterol and decreased risk of myocardial infarction (MI) and lower Alzheimer’s disease (AD) at rs429358 in *APOE* (38). In contrast, rs7412 in *APOE* did not associate with hepatic steatosis, showing the allelic complexity of this gene (38).

Several studies have used ICD codes for MASLD alone, which have identified genes also seen in imaging-based studies, e.g. *PNPLA3*, *TM6SF2*, mitochondrial amidoxime-reducing component 1 (*MARC1*), *GCKR*, tribbles homolog 1 (*TRIB1*), FTO α-ketoglutarate dependent dioxygenase gene (*FTO*), and *APOE* (30, 31). Following this report, Haas et al. (32) extended this resource by developing a machine-learning algorithm to accurately estimate liver fat using raw abdominal MRI. As a result, the sample size increased to 36,703 UK Biobank participants. They identified associations at *PNPLA3*, *TM6SF2,* and *APOE* as well as in alcohol dehydrogenase 1B (class I), β polypeptide gene (*ADH1B*), the microtubule associated serine/threonine kinase 3 gene (*MAST3*), and the mitochondrial amidoxime reducing component 1 gene (*MTARC1*). *MAST3,* as discussed below, may be better described as an abdominal obesity–affecting gene, suggesting that estimation of liver fat may have been driven by abdominal obesity predictors in the algorithm.

Whole-exome sequencing may provide additional insights into disease biology (42). This approach can identify rarer variants than is typically feasible with genotyping arrays, and it is possible to associate groups of rare variants with traits by summing their burden (gene-based testing) (52). Taking this approach, Verweij et al. (42) conducted gene-based tests for ALT, aspartate aminotransferase (AST), and liver diseases, and identified five suggestive genes, apolipoprotein B (*APOB)*, *ABCB4*, *SLC30A10*, and *TM6SF2*, associated with increased liver disease, and cell death inducing DFFA like effector B (*CIDEB*), associated with decreased risk of liver disease. The authors then assessed rare predicted loss-of-function *CIDEB* variants in patients undergoing bariatric surgery and liver biopsy; individuals with these rare variants were less likely to have steatosis, steatohepatitis, or fibrosis (42).

Imaging can be combined with diagnosis codes for MASLD, e.g. ICD-10 K76.0 and/or K75.81, to further increase power. Using this approach, Sveinbjornsson et al. (41) metaanalyzed MRI-PDFF with ICD codes and implicated three additional suggestive loci in disease, i.e., patatin like phospholipase domain containing 2 (*PNPLA2*), the transmembrane channel-like protein 4 gene (*TMC4* near *MBOAT7* [membrane-bound O-acyltransferase 7]), and the torsin family 1 member B gene (*TOR1B*). (Notably, *MBOAT7* was initially identified to be associated with alcohol-related cirrhosis (45) and subsequently implicated in MASLD in candidate gene studies; refs. 53, 54).) They identified the lysosomal hydrolase β-glucuronidase gene and homeostatic iron regulator gene (*HFE*) as suggestive MASLD-associated variants. Other suggestive loci included microsomal TG transfer protein large subunit (*MTTP*), apolipoprotein H (*APOH*), and cordon-bleu WH2 repeat protein like 1 (*COBLL1*) (41). Of note, the *HFE* variant identified is the primary variant responsible for hereditary hemochromatosis that is associated with hepatic iron content; as discussed above, markedly increased iron content can falsely increase MRI-PDFF (55). More recently, Chen et al. (28) combined CTs from multiethnic population-based cohorts from GOLD with MRI-PDFF in UK Biobank and diagnostic-code-assessed MASLD to perform the largest metaanalysis to date. Their analyses identified 17 loci associated with MASLD including in *PNPLA3, TM6SF2, GCKR, APOE, MTARC1, PNPLA2, MBOAT7, TORB1, ADH1B, MTTP, GPAM, FTO, TRIB1*, and *COBLL1/GRB14* (growth factor receptor-bound protein 14) and findings at the insulin receptor gene (*INSR*), protein tyrosine phosphatase receptor type D gene (*PTPRD*), and sterol regulatory element binding transcription factor 1 gene (*SREBF1*).

Beyond direct measures of liver fat, published reports have used ALT as a proxy phenotype to identify MASLD-associated variants, which allows for a rapid increase in sample size and study power compared with imaging or histology. In 2021, Chen et al. (18) metaanalyzed samples from UK Biobank and BioBank Japan to perform a GWAS of liver enzyme concentrations and subsequently validated associated variants using CT-measured liver attenuation. This analysis identified 21 suggestive loci including 1-acylglycerol-3-phosphate O-acyltransferase 5 gene, *TRIB1*, collagen type IV alpha 2 chain gene, ER lipid raft associated 1gene, mitochondrial glycerol-3-phosphate acyltransferase (*GPAM*), ligand dependent nuclear receptor corepressor-like gene, peptidase D and a non-coding RNA, LOC102723704. However, only 21 of the 172 ALT-increasing variants were associated with increased hepatic steatosis at even nominal significance (*P <* 0.05). More recently, Vujkovic et al. (15) identified 77 genome-wide significant loci associated with ALT using data from the Million Veterans Program (MVP). However, only 17 were subsequently implicated in MASLD using histologic and image-based cohorts. These included the suggestive *MTTP*, *APOH*, *COBLL1*, *FTO*, IL-1 receptor antagonist gene, PPAR-γ gene, lysosomal thiol reductase gene, and genome-wide significant serpine family A member 1 gene (*SERPINA1*). The observation in both studies that only a subset of the ALT variants translate to more precise measures of MASLD suggests that current MASLD studies are underpowered or that ALT represents pathophysiologic mechanisms beyond fat accumulation.

Finally, some studies developed scores based on clinical data to diagnose MASLD to increase statistical power and then conducted case-control analyses based on predicted MASLD to identify dozens of variants (35–37). However, when applied in the general population, these scores can detect diseases correlated with steatosis, but which are not direct measures of steatosis, such as obesity, waist-hip ratio, and diabetes/insulin resistance. For example, two of these studies of MASLD predicted based on complex scores have identified variants in *GRB2* and *MAST3,* which are known strong waist-hip ratio altering loci (56).

To summarize the biological context of reproducible steatosis-associated genes from the above studies, we extracted variants associated with MASLD at genome-wide significance levels (*P < 5* × 10^–08^) (Table 3). We included studies that assessed genetic associations with hepatic steatosis using imaging, histology, or ICD codes. We used a 500 Kb distance criteria cutoff to determine independent hits, except for *ADH1B* and *MTTP,* which were both included at less than 500 Kb distance as they have been reported as independent loci (28). If multiple SNPs were reported for a gene, we chose a single most-cited representative SNP. The genes implicated from those associations and their biology are shown in Table 3. Overall, these fall into groups of genes that affect lipoprotein input or output, glucose metabolism, adiposity/fat distribution, insulin resistance, or mitochondrial/ER biology.

### Steatohepatitis and fibrosis/cirrhosis.

This Review focuses on hepatic steatosis, but we briefly discuss other MASLD phenotypes, i.e., steatohepatitis and fibrosis. The genetics underlying histologically confirmed steatohepatitis or fibrosis have been less well characterized than for steatosis largely due to limited statistical power. In one early study from the Nonalcoholic Steatohepatitis Clinical Research Network (NASH CRN), a variant in *FDFT1* associated with MASLD activity score, another intergenic chromosome 7 variant with fibrosis, and variants in *COL13A1*, *LTBP3*, and *TFCAB4B* with lobular inflammation at *P <* 1 × 10^–06^ (26). Another NASH CRN study also found nominally significant (*P <* 0.05) associations between *PNPLA3* genotype and fibrosis or lobular inflammation (57). Anstee et al. also found that the *PNPLA3* variant and a *LEPR* variant associated with MASH at genome-wide significance (25). Namjou et al. also included case-only GWAS (*n =* 235) and identified novel associations between MASLD activity score and an *IL17RA* variant (as well as the known association with *PNPLA3* genotype) and between fibrosis stage and two intergenic loci (annotated to ZFP90-CDH1 and FABP1) (37).

To overcome the power limitations of histologic analyses, Parisinos et al. also conducted GWAS for corrected T1 time (cT1), an MRI-derived biomarker for fibroinflammation that correlates with histologic steatohepatitis, in 15,538 individuals (39). Another study by Andersson et al. found that cT1 is better than liver fat content at identifying MASH patients at higher risk of disease progression (58). Parisinos et al. identified variants in metal transporter genes (*SLC30A10*, *SLC39A8*) as well as in *PNPLA3* and *TM6SF2* as associated at genome-wide significance with cT1 and aminotransferases (39). The variant at the *SLC39A8* gene, however, was later shown to not have an increased risk of liver disease and likely represents the MRI picking up liver manganese, again suggesting that these indirect measures of MASH can pick up other unintended phenotypes (59). Given that biopsy is becoming less frequently used in clinical practice, identifying better imaging biomarkers of steatohepatitis and fibrosis and expanding imaging-based cohorts may be the most realistic way to increase our understanding of the genetic basis of steatohepatitis and fibrosis.

Several GWAS of cirrhosis have been conducted; however, most of these were not focused on MASLD-related cirrhosis, but rather included cirrhosis from any etiology (60–64). Many steatosis-increasing variants were also associated with cirrhosis, including those in *PNPLA3*, *TM6SF2*, and *MBOAT7* at a genome-wide level of significance, and suggestive at *APOE*, *HSD17B13*, *MARC1*, and *SERPINA1* (45). Ghouse et al. conducted one of the largest recent studies, which included derivation and validation sets of nearly 40,000 cirrhosis cases and over 2,000,000 controls (63). This study identified and validated 14 variants associated with cirrhosis at genome-wide significance including variants in/near *PDE4B*, *ZFP36L2/HAAO*, *GYPC*, *TRIB1*, *GPAM*, and *ALDH2*. They also queried effects of liver enzyme–increasing variants on cirrhosis and found an additional 21 variants at a false discovery rate of less than 0.05. Of note, some of these variants were associated with increased hepatic steatosis including many of those described earlier, whereas others had no such association, including the *HSD17B13* variant. These findings suggest that some genetic variants may promote fibrosis/cirrhosis by promoting steatosis whereas others increase risk of fibrosis/cirrhosis through mechanisms not related to steatosis, and still others may have both effects.

## PRSs

**Figure 1:**
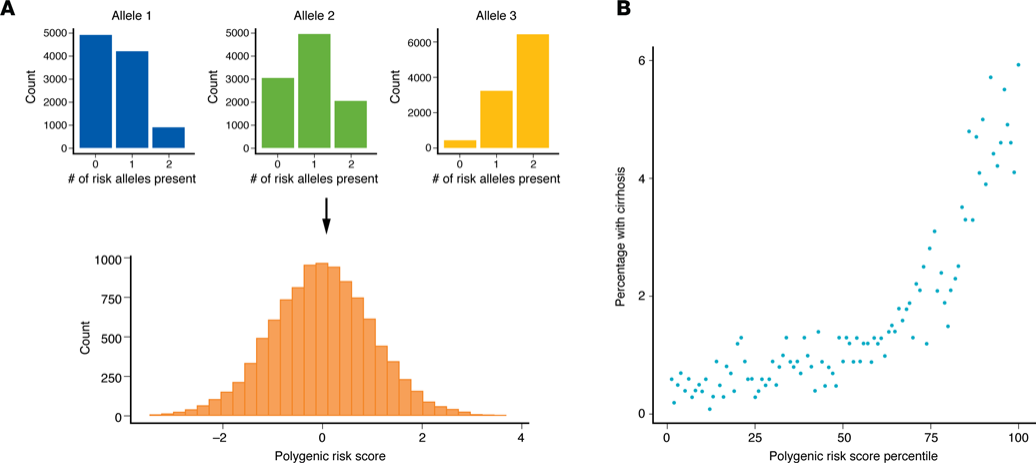
PRSs. (**A**) Sample distribution of risk alleles, which when combined and weighted by effect size, can contribute to calculation of a continuous PRS. (**B**) Sample PRS plotted versus the percentage of individuals with cirrhosis to show how this score can identify some individuals with high risk of developing the disease.

Above, we described variants that have been associated with MASLD. However, single variants may not adequately capture the overall genetic risk of an individual. For example, a person carrying one *PNPLA3*-rs738409-G risk allele who also carries two *TM6SF2*-rs58542926-T risk alleles is presumably at higher risk than someone with the same *PNPLA3* genotype who carries no *TM6SF2* risk alleles. Therefore, more recently there has been interest in PRSs to better quantify genetic risk. PRSs typically sum the number of risk alleles that each person carries. PRSs can be unweighted, i.e. number of risk alleles, or weighted so that alleles that have a stronger effect on steatosis are weighted more heavily. Thus, PRSs essentially convert genotypes (categorical variables) into a numerical score (continuous variable) (Figure 1).

Multiple studies have evaluated the impact of MASLD-associated PRSs and their effects on clinically-relevant endpoints such as MASLD, NASH, cirrhosis, and HCC (Table 4). PRSs for MASLD are associated not only with markedly increased risk for MASLD among patients in the general population, but also an increased risk of advanced fibrosis, cirrhosis, or HCC in the general population and among patients with MASLD (Table 4). Limitations of using PRSs to calculate individual risk include variant heterogeneity, weighing of risk scores, and choice of population. Further, more complex scores do not necessarily demonstrate higher performance than simpler scores (63, 65). Finally, the vast majority of the studies identifying genetic variants include participants of European descent, so whether these scores accurately represent genetic variation and populations remains to be determined.

## Gene-environment interactions

Genetic risk is not usually fixed, but dependent on environmental precipitants to cause disease. For example, an individual with normal weight and minimal insulin resistance may have only a 10% increased risk of having hepatic steatosis based on the *PNPLA3*-rs738409-CG genotype (vs. CC genotype), whereas an individual with diabetes and obesity may experience a 50%–100% increase in risk from the same genotype.

**Figure 2:**
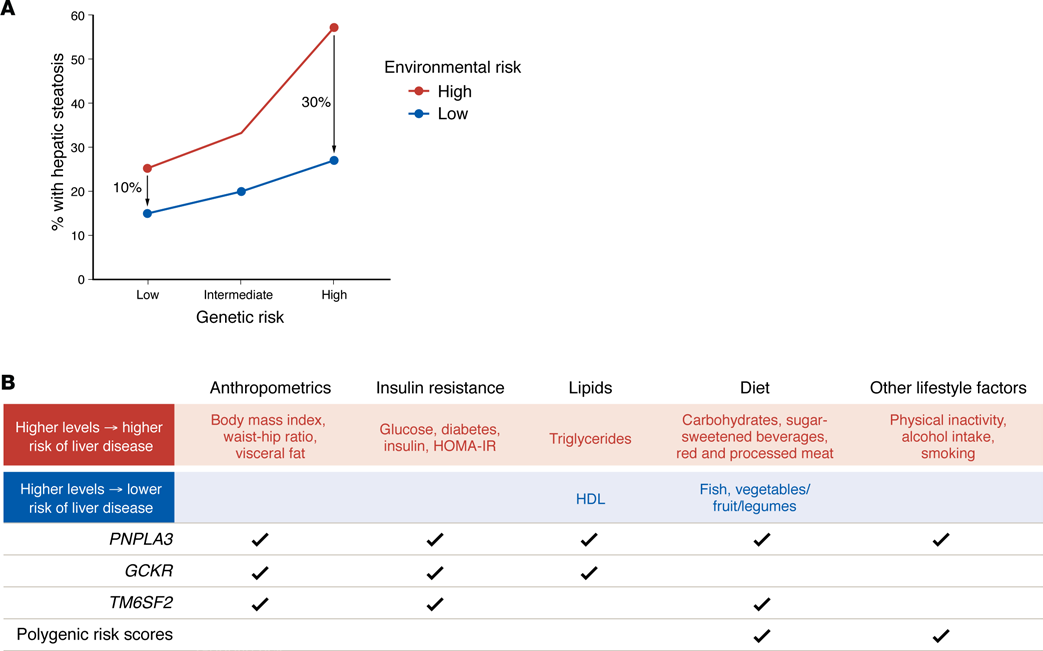
Gene-environment interactions. (**A**) Schematic of gene-environment interactions. In this hypothetical example, the prevalence of hepatic steatosis (*y* axis) in individuals with low (red) vs. high (blue) environmental risk increases in a dose-dependent manner based on genetic risk (*x* axis). However, the effect of environmental risk is much greater in those with low genetic risk (absolute difference 10%) versus high genetic risk (absolute difference 30%), indicating a gene-environment interaction. (**B**) Summary of reported gene-environment interactions for hepatic steatosis severity or liver-related complications in MASLD. The leftmost column lists genes whose variants are known to interact with environmental risk. The top row displays categories of environmental risk factors that interact with genetic risk. Environmental risk factors in red indicate that higher levels of the risk factor confer *greater* risk of liver disease in those with higher genetic risk, whereas risk factors in blue indicate that higher levels of the risk factor confer *lower* risk in those with higher genetic risk. Checkmarks show where there is evidence for interactions between specific genes or the polygenic risk score with categories of environmental factors.

The literature on gene-environment interactions is strongest for *PNPLA3* (66). The *PNPLA3* genotype interacts with both nonmodifiable (age, sex, genetic ancestry) and modifiable risk factors (visceral adiposity, obesity, insulin resistance, lipids) to multiplicatively increase risk of hepatic steatosis (66) (Figure 2). *PNPLA3* interacts strongly with insulin resistance to drive hepatic steatosis, and a combination of the *PNPLA3* risk allele, insulin resistance, and their interaction explained 8% of the variation in hepatic steatosis in nondiabetic individuals, suggesting that many individuals at high risk of hepatic steatosis are not currently being treated (67). Gene-environment interactions are relevant not only for MASLD, but for the presence of fibrosis (68) and development of other disease endpoints. Notably, the *PNPLA3* genotype strongly interacts with diabetes and advanced fibrosis to drive risk of hepatic decompensation, for example (69, 70).

Even more readily modifiable factors may interact with genetic risk. Chen et al. found that individuals with *PNPLA3* risk alleles who followed a Mediterranean-style diet or had high intake of fruits, vegetables, and legumes derived even more benefit in hepatic steatosis reduction attributable to these dietary patterns than those without the alleles; a PRS additionally interacted with fish intake to strengthen its protective effects against steatosis (71). The *PNPLA3* genotype also interacts with meat intake, carbohydrate intake, smoking, and sugar-sweetened beverage intake to exacerbate the deleterious effects on steatosis of these dietary patterns (72–76). Ge et al. found that a PRS for liver disease interacted with total physical activity and sedentary time, such that individuals at high genetic risk also experienced the greatest absolute reduction in MASLD risk from high physical activity and low sedentary time (77). Vilar-Gomez et al. also found that the *PNPLA3* genotype interacted with light alcohol intake and high cholesterol intake to markedly increase risk of liver-related death in a population-based cohort (78).

The literature on other gene-environment interactions for other individual genetic variants is more limited. One recent study found that the *TM6SF2* genotype associated with red/processed meat intake (71), and another found that the *GCKR* genotype associated with insulin/insulin resistance and TGs to multiplicatively increase hepatic steatosis (67).

## Clinical outcomes

One major issue limiting clinical applicability of genetics for risk stratification is whether genetics improve upon existing tests. The literature on this topic has been mixed and depends largely on the population evaluated. One recent study in a community-based cohort (UK Biobank) evaluated the diagnostic accuracy of noninvasive tests, with or without addition of a PRS for cirrhosis (79). They found that the highest-performing noninvasive tests, namely AST–to–platelet ratio index (APRI), MASLD fibrosis score, and Fibrosis-4 (FIB4) score, had an area under the receiving operator characteristic curve (AUROC) of around 0.8, but addition of PRS to the models did not improve on the performance characteristics of these tests.

In contrast, others have found that PRSs improve predictive power from clinical risk factors. One recent study in the UK Biobank found that a PRS was not associated with cirrhosis and HCC among patients with low MASLD fibrosis score, FIB4, and APRI (80). However, in patients with intermediate or high noninvasive scores, diabetes, or obesity — all risk factors for advanced disease in MASLD — PRSs were strongly associated with incident cirrhosis and HCC (80). Another study of the Michigan Genomics Initiative and UK Biobank participants with elevated ALT found that in low FIB4 individuals, genetics were weakly or not associated with incident severe liver disease, but individuals with intermediate FIB4 but high-risk genetics (*PNPLA3*-rs738409-GG genotype) and cardiometabolic disease (diabetes) had risk comparable to that of high FIB4 individuals (70). Another study found that adding a PRS to FIB4 alone resulted in less misclassification of at-risk patients with low FIB4 compared with FIB4 alone (81). The FIB4 category may also influence associations between genetic risk and extrahepatic outcomes including cardiovascular disease (CVD) (82).

Genetics carries utility beyond histologically defined MASLD also. The *PNPLA3* genotype is associated with major adverse liver outcomes independently of histologic fibrosis stage. In the MASH Clinical Research Network, the *PNPLA3*-rs738409-G allele was associated with increased risk of liver-related outcomes, with subhazard ratio 1.51 and 1.94 for CG and GG genotypes versus CC, respectively (69). A multicenter cohort of 1,178 patients with biopsy-confirmed MASLD in Japan also showed associations between the *PNPLA3* genotype and liver-related outcomes (83). Notably, in both cohorts, the absolute effect of the *PNPLA3* genotype was far greater in participants with advanced fibrosis versus those without.

These disparate reports on whether PRSs improve on clinical predictors are caused by differences in several factors. First, the better the performance of clinical predictors in a given population, the lower the incremental benefit of genetics. Second, genetics typically add about 0.02 points to the AUROC and the effect of the genetics has an odds ratio or hazard ratio of greater than 2. Authors can report these effects as large or small depending on author perspective. Third, the frequency of risk variant(s) heavily influences power to detect an effect. Finally, due to gene-environment interactions, the prevalence of underlying clinical risk factors, such as elevated FIB4, obesity, or diabetes, can greatly increase the effects of genetic risk. We note that PRSs are associated with clinically relevant outcomes, but choosing the correct cohort (i.e., those at intermediate to high pretest probability of disease) is key to identifying clinically actionable risk stratification using genetics.

## Molecular subtyping, outcomes, and precision therapy

Genetic analyses suggest that there are multiple causes of MASLD. With a better understanding of these molecular causes comes the opportunity to better tailor care. Because some genetic causes of MASLD also associate with alcoholic liver disease, this has led the field to moving away from artificial distinctions of nonalcoholic versus alcoholic liver disease towards a more unified steatotic liver disease designation. Further, the finding that many of the genetic variants that affect lipid or glucose biology anchor the disease in disruption of metabolic processes has led to a changing of the name of the disease to “metabolic dysfunction–associated steatotic liver disease.” The revised disease terminology therefore now includes MASLD, MASLD and increased alcohol intake (MetALD), and alcohol-related liver disease under a single umbrella definition (1). Understanding the pathophysiology and genes to target to mitigate disease risk will be important to inform precision treatments (84, 85). For example, knowing whether an individual has iron or B12 deficiency causing anemia can help identify effective therapies with few side effects such as oral iron or B12 to help reverse the disease, rather than blood transfusions. In this same way, knowing the biology of MASLD genetic variants can help us to identify who will develop liver cirrhosis versus who will develop MASLD-related metabolic disease such as heart disease or diabetes.

Researchers have proposed subdividing MASLD-associated genetic variants based on their pleiotropic patterns and/or putative pathophysiologic mechanisms. One group proposed a paradigm (28) whereby partitioned PRSs affect outcomes differently and illustrate how this can help explain the heterogeneity of metabolic diseases seen in MASLD patients. Clusters of variants with specific functional associations with MASLD include subgroups labeled low liver lipoprotein out, high lipoprotein in, low lipid burn, insulin, absorption, glucose, and diversion.

**Figure 3:**
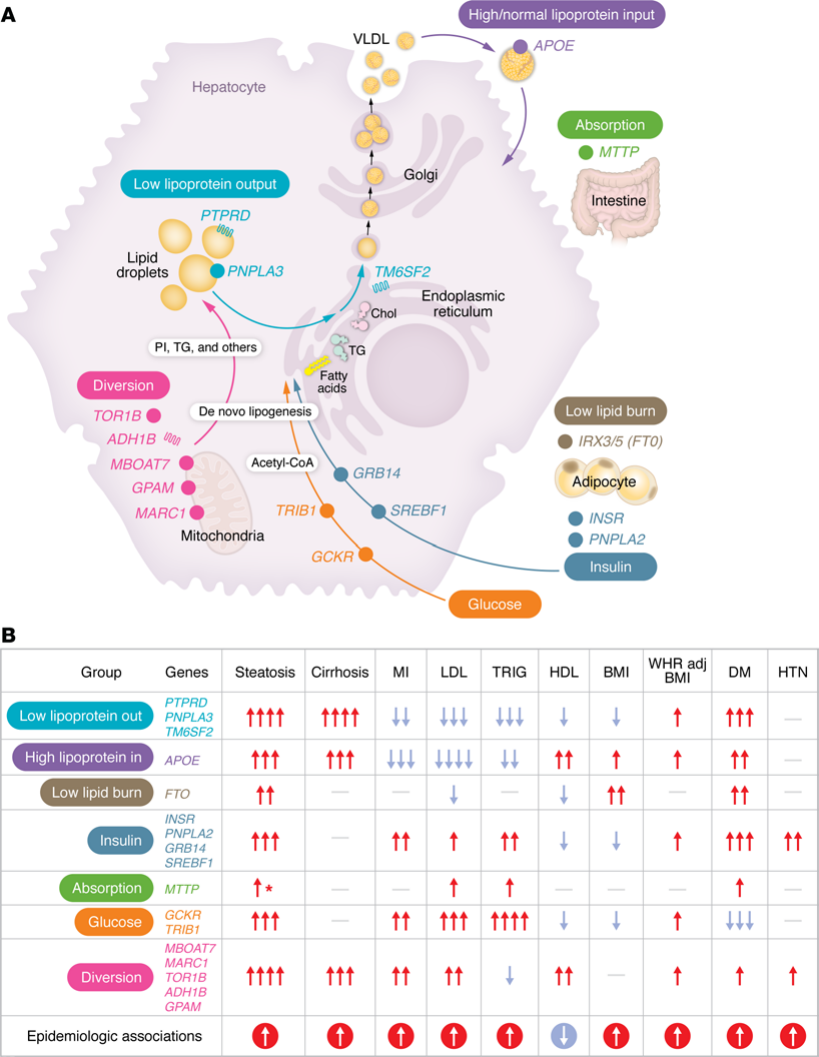
Risk gene subgroups associated with PheWAS-identified phenotypes. Panel (**A**) illustrates the subgroups of risk genes in the context of intracellular and systemic functions linked to their gene products. Panel (**B**) summarizes the phenotype effects (top row) associated with each risk gene subgroup (leftmost column) that were identified in previously reported PRSs based on human outcomes. Effect sizes for continuous traits are reported as for β values on rank-based inverse normally transformed traits, and as log odds ratio for dichotomous traits. PRSs with significant positive associations are shown as red up arrows, those with significant negative associations are shown as blue down arrows, and those with no significant association (*P* > 0.05), as hyphens. Effect sizes for continuous traits are reported as β values on rank-based inverse normally transformed traits, and as log(odds ratio) for dichotomous traits. One, two, three, or four arrows indicate absolute value of effect size of <0.04, 0.04-<0.08, 0.08-<0.16, or ≥0.16, respectively. Epidemiologically-expected associations are shown at the bottom and the arrows are agnostic to effect size. VLDL, very low-density lipoproteins; TRIG, triglycerides; WHRadjBMI, waist-hip ratio adjusted for BMI; DM, diabetes mellitus; HTN, hypertension. Figure adapted from ref. 28 with permission from Springer Nature, which retains the rights to the reference image. MTTP effect on steatosis based on meta-analysis with additional cohorts beyond UK Biobank.

The gene groupings from Chen et al. (28) are summarized in Figure 3 ([A] genes highlighted and [B] their PheWAS associations) and described in detail below: they include low liver lipoprotein out, high lipoprotein in, low lipid burn, insulin, absorption, glucose, and diversion.

In the low liver lipoprotein out group, the MASLD-promoting alleles at *PNPLA3*, *TM6SF2*, and *PTPRD* all decrease TGs and LDL cholesterol while increasing cellular TG and cholesterol burden in the liver by several mechanisms. Some alleles may decrease liver lipoprotein output (*TM6SF2*), perhaps by interfering with the function of APOB (86), and others (*PNPLA3*, *PTPRD*) by affecting lipid droplet biology to decrease release of TG (43, 44). In terms of outcomes, this group of genes increases cirrhosis risk, decreases MI, increases diabetes, and decreases BMI.

A second subgroup, identified as the high lipoprotein in group and characterized by *APOE* variants, increases return of lipoproteins to the liver (87) and in this way may promotes MASLD. Similar to the lipoprotein out group, this subgroup decreases TG and LDL. It increases cirrhosis risk, decreases MI, and increases diabetes and BMI.

The low lipid burn group may cause disease by preventing the use of lipids to produce energy. The *FTO* region (involving interactions with *IRX3/5*, encoding Iroquois 3/5) (88) decreases LDL with little effect on TG. This group of alleles increases BMI and diabetes while not having much effect on increasing cirrhosis or MI. These may promote hyperalimentation in one way or another to increase adiposity and diabetes to promote disease.

The insulin subgroup may promote lipodystrophy/insulin resistance. Variants identified in *GRB14/COBLL1*, *PNPLA2*, *SREBF1*, and *INSR* all increase LDL and TG. They have no effect on cirrhosis and increase MI and diabetes while decreasing BMI. Variants in this group may result in these phenotypes due to the role of the genes in promoting subcutaneous fat storage; hence, disruption of their function can cause lipodystrophy and insulin resistance. This in turn may promote MASLD via increased release of fatty acids from adipose tissue (89, 90) or increased hepatic de novo lipogenesis in liver (91). Variants in *INSR* and loss of *GRB14*, a negative regulator of insulin signaling, may directly increase insulin action on the liver to promote de novo lipogenesis. Indeed, insulin promotes the synthesis of TG via upregulation of the master transcriptional regulator *SREBF1* (92). *PNPLA2* is the major protein in adipose tissue that normally promotes release of fatty acids from adipose tissue to increase their delivery to liver, promoting MASLD. Loss of *PNPLA2* in mice prevents SREBP activation and de novo lipogenesis in liver as at least one mechanism by which *PNPLA2* may affect MASLD (93).

The absorption subgroup consists of *MTTP,* which functions to package lipoproteins in the intestine and liver, and rare mutations in this gene cause abetalipoproteinemia (94). The global effects of this locus on phenotypes include increasing TG, LDL, and diabetes but not having significant effects on BMI, MI, and cirrhosis. The effects seen in the PheWAS are better explained by a global increase or decrease in fatty acid absorption at the level of the intestine rather than an effect at the level of the liver, which would be expected to result in a PheWAS pattern more like *TM6SF2*.

A glucose subgroup may convert glucose to TG. *GCKR* and *TRIB1* all increase serum TG and LDL. They do not have an effect on promoting cirrhosis but rather increase risk of MI, while decreasing diabetes and BMI. One mechanism by which *GCKR* and *TRIB1* may promote MASLD is by utilizing glucose to make fatty acids by de novo lipogenesis (95, 96).

The diversion subgroup diverts TG to phospholipids and other lipids. The MASLD-promoting alleles at *GPAM*, *MARC1*, *TOR1B*, *MBOAT7*, and *ADH1B* all increase LDL and decrease TG, thus, diverting TG from being excreted by the liver and retaining them in one way or another to cause pathology. The alleles at these genes may serve to divert carbons from TG to cholesterol, phospholipids, glycerolipids, and other metabolites. GPAM may do this directly, as it is the rate-limiting mitochondrial enzyme in the formation of TG (97–99). *MARC1* may affect phosphatidylcholine metabolism to affect MASLD (100). *MBOAT7* may promote MASLD by diverting TG to accumulation of lysophosphatidyl inositol (101). ADH1B metabolizes many substrates including ethanol to promote hepatic steatosis (102, 103). Variants in this subgroup of genes increase risk of cirrhosis, MI, and diabetes while being neutral on BMI. How the other variants identified by GWAS for MASLD from other papers relate to effects in these subgroups remains to be determined.

Separate recent studies have divided MASLD-promoting variants in other ways. Ahmed et al. classified variants as those that promote hepatic steatosis via de novo lipogenesis or via impaired hepatic fat export (104). Here, the variants associated with increased de novo lipogenesis are strongly associated with CVD and diabetes and weakly with increased risk of advanced liver disease (cirrhosis and HCC). In contrast, the variants associated with impaired hepatic fat export were associated with larger effects on advanced liver disease, but decrease or have no effect on CVD (104). In this study, de novo lipogenesis–promoting variants roughly corresponded to the “glucose” group in the above study (*TRIB1*, *GCKR*) as well as *ADH1B* and *CDHR4* variants, while those that impaired hepatic fat export (*PNPLA3*, *TM6SF2*, *APOE*, *SUGP1*) corresponded roughly to the low lipoprotein output and high/normal lipoprotein input groups from the Chen study (28). A third group of variants that roughly corresponded to the “diversion” group detailed above had little to no effect on CVD. Separately, Jamialahmadi et al. conducted GWAS for hepatic steatosis or cT1 adjusted for anthropometrics and divided steatosis/cT1-promoting variants into those that increased (concordant) versus decreased (discordant) serum TGs (105). Similar to the Ahmed et al. study, concordant variants had large effects on cardiometabolic disease and an increase in liver-related outcomes, while the discordant group (dominated by *PNPLA3* and *TM6SF2* variants) had larger effects on HCC and cirrhosis but no effect or even protection from CVD (105). Overall, there was much congruency in the subtyping between these three studies.

## Future directions

We highlight several key future directions for genetic research in MASLD and how it may inform science and clinical practice. (a) Expanding diversity of populations studied. Studies of more rare variant effects and effects in ancestries beyond Europeans will likely identify more variants and genes that affect MASLD. (b) Disease heterogeneity and health-related outcomes. MASLD PRS subtypes help explain the heterogeneity of metabolic phenotypes seen in MASLD patients. For example, low lipoprotein out, high lipoprotein in, and diversion groups predispose to cirrhosis whereas the other groups do not. Analogously, the insulin, glucose, and diversion groups predispose to MI, whereas other groups do not. By knowing a person’s risk subtype, we might be able to better predict their outcomes and guide patient recommendations toward precision medicine in the future. (c) Combining with clinical risk factors. Relatively few studies have assessed incremental impact of genetic variants beyond frequently used clinical predictors, and fewer have studied the increasingly-utilized elastography-based noninvasive tests (106). (d) Implications for treatment. With increasing molecular targeting of these genes for therapeutic purposes (107, 108), we can expect better outcomes when treatment matches etiology and possibly worse side effects when there is mismatch of treatment with subtype. For example, a treatment reversing the effects of PNPLA3 may increase TG and LDL as well as risk of MI, which is most relevant in those at high baseline risk such as those in the insulin group.

## Author contributions

VLC was the lead for phenotype and polygenic scores and critically reviewed the manuscript. AK was the lead for paper formatting and critically reviewed the manuscript. AO critically reviewed the manuscript. YC critically reviewed the manuscript. PP engaged in literature review and critically reviewed the manuscript. PBP critically reviewed the manuscript. NDP was the lead for genomic methods, reviewed the literature, and critically reviewed the manuscript. EKS designed the concept, did original drafting of all sections of manuscript, was the lead for organization of the project, engaged in biological interpretation, subtyping, and precision therapy, and critically reviewed the manuscript.

## Figures and Tables

**Table 4:**
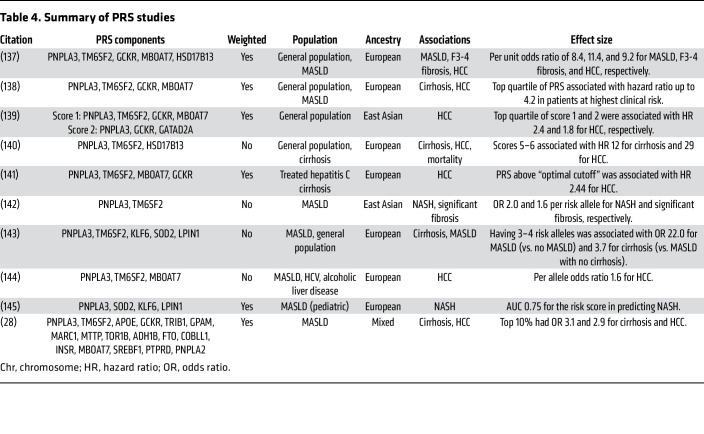
Summary of PRS studies

**Table 3:**
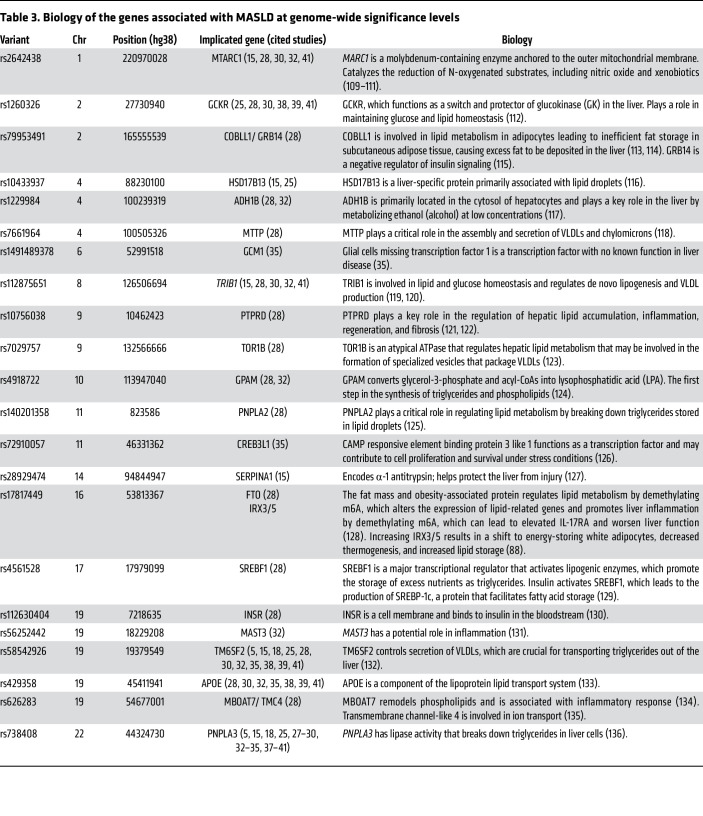
Biology of the genes associated with MASLD at genome-wide significance levels

**Table 2:**
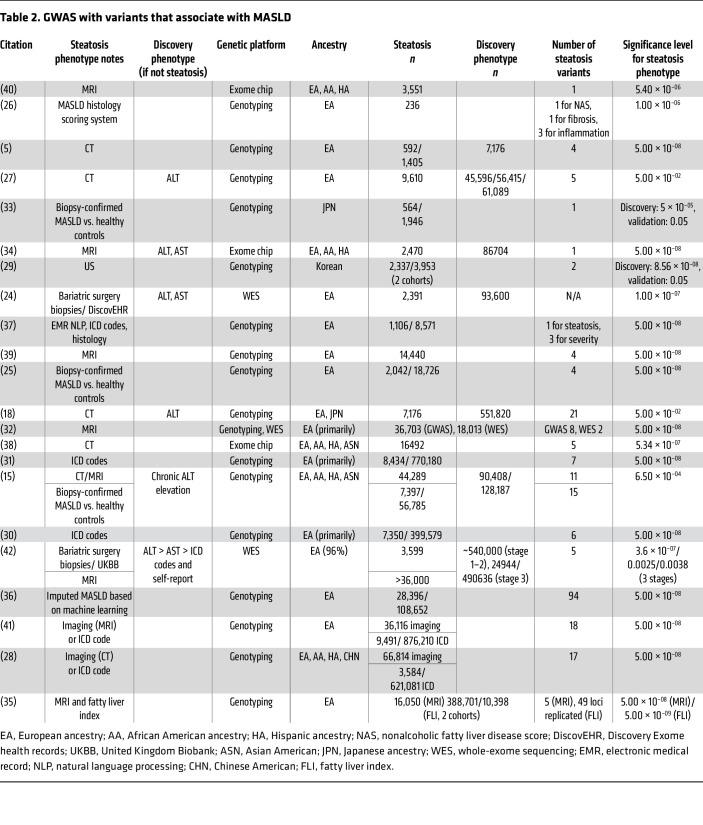
GWAS with variants that associate with MASLD

**Table 1:**
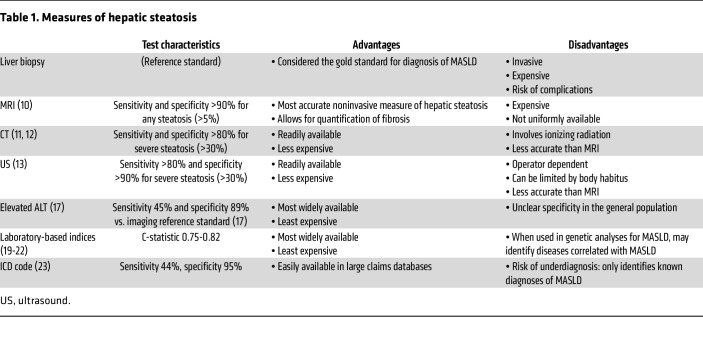
Measures of hepatic steatosis

## Version 1 04/01/2025

Electronic publication
